# Structure and Dynamics of the Gut Bacterial Community Across the Developmental Stages of the Coffee Berry Borer, *Hypothenemus hampei*

**DOI:** 10.3389/fmicb.2021.639868

**Published:** 2021-07-01

**Authors:** Fernan Santiago Mejía-Alvarado, Thaura Ghneim-Herrera, Carmenza E. Góngora, Pablo Benavides, Lucio Navarro-Escalante

**Affiliations:** ^1^Department of Entomology, National Coffee Research Center (Cenicafe), Manizales, Colombia; ^2^Departamento de Ciencias Biológicas, Universidad Icesi, Cali, Colombia

**Keywords:** coffee berry borer, microbiota, symbionts, gut, bacteria, coffee

## Abstract

The coffee berry borer (CBB); *Hypothenemus hampei* (Coleoptera: Curculionidae), is widely recognized as the major insect pest of coffee crops. Like many other arthropods, CBB harbors numerous bacteria species that may have important physiological roles in host nutrition, detoxification, immunity and protection. To date, the structure and dynamics of the gut-associated bacterial community across the CBB life cycle is not yet well understood. A better understanding of the complex relationship between CBB and its bacterial companions may provide new opportunities for insect control. In the current investigation, we analyzed the diversity and abundance of gut microbiota across the CBB developmental stages under field conditions by using high-throughput Illumina sequencing of the 16S ribosomal RNA gene. Overall, 15 bacterial phyla, 38 classes, 61 orders, 101 families and 177 genera were identified across all life stages, including egg, larva 1, larva 2, pupa, and adults (female and male). Proteobacteria and Firmicutes phyla dominated the microbiota along the entire insect life cycle. Among the 177 genera, the 10 most abundant were members of *Ochrobactrum* (15.1%), *Pantoea* (6.6%), *Erwinia* (5.7%), *Lactobacillus* (4.3%), *Acinetobacter* (3.4%), *Stenotrophomonas* (3.1%), *Akkermansia* (3.0%), *Agrobacterium* (2.9%), *Curtobacterium* (2.7%), and *Clostridium* (2.7%). We found that the overall bacterial composition is diverse, variable within each life stage and appears to vary across development. About 20% of the identified OTUs were shared across all life stages, from which 28 OTUs were consistently found in all life stage replicates. Among these OTUs there are members of genera *Pantoea*, *Erwinia*, *Agrobacterium*, *Ochrobactrum*, *Pseudomonas*, *Acinetobacter*, *Brachybacterium*, *Sphingomonas* and *Methylobacterium*, which can be considered as the gut-associated core microbiota of *H. hampei*. Our findings bring additional data to enrich the understanding of gut microbiota in CBB and its possible use for development of insect control strategies.

## Introduction

Mutualistic bacteria and fungi provide advantageous services to insect hosts by facilitating the digestion of recalcitrant food (Genta et al., 2006; Brune, 2014), providing essential nutrients (Feng et al., 2019), promoting immunity or protection against pathogens, parasites, or predators (Koch and Schmid-Hempel, 2011; Muhammad et al., 2019), contributing to inter- and intraspecific communication (Engl and Kaltenpoth, 2018; Calcagnile et al., 2019), and modulating the interaction of phytophagous insects with host plants (Acevedo et al., 2017). This complex mutualistic relationship between microbial symbionts and insect hosts has likely played a special role in the adaptive radiation and diversification of phytophagous insect species due to the potential microbial influence on host plant-associated ecological opportunity and divergent natural selection (Janson et al., 2008).

The understanding of the interaction between insects and their gut-associated symbionts has a key relevance in agriculture due to the potential application of this knowledge for the management of insect pests. For example, obligate symbionts required for insect survival are potential targets for suppression of insect pest populations by disruption of the symbiont-host relationship (Douglas, 2007). Insect gut symbionts can also be genetically manipulated (paratransgenesis) and used as vehicles for the delivery of effector molecules that negatively affect insect survival or fitness (Whitten et al., 2016). Furthermore, the identification of insect gut symbionts capable of enhancing insecticide resistance in several insect species (Kikuchi et al., 2012; Xia et al., 2018) is gaining interest for the monitoring and management of chemical insecticide resistance (Cheng et al., 2017). An increasing number of agriculturally relevant pest insects are now the focus for microbiome-insect mutualism studies thanks to the development of culture-independent techniques such as gene amplicon high-throughput sequencing (e.g., 16S rRNA) and shotgun metagenomics (Bharti and Grimm, 2019; Gurung et al., 2019).

The Coffee Berry Borer (CBB); *Hypothenemus hampei* (Coleoptera: Curculionidae) is a notorious pest of coffee (*Coffea* spp.) worldwide. The CBB female directly attacks coffee fruits, where it feeds and builds galleries inside the seed for the full development of the insect life cycle (Damon, 2000). Mating occurs within the infested fruit between siblings, which causes high endogamy. Fertilized females leave the infested fruit and look for new coffee fruits to start the cycle again. Previous studies based on culture-independent techniques revealed that the CBB female gut contains a broad diversity of bacteria, mainly composed by the Phylum Proteobacteria, and to a lesser extent Firmicutes and Bacteroidetes (Ceja-Navarro et al., 2015; Mariño et al., 2018). The overall microbiota structure in the CBB female is variable and influenced by the coffee-host species diet and geographic origin of the insect population; nonetheless, a common core bacteria is shared among insects from different geographic locations (Ceja-Navarro et al., 2015). This core seems to be constituted by species within the genera *Pantoea*, *Erwinia*, *Agrobacterium*, *Ochrobactrum*, *Pseudomonas*, *Acinetobacter*, *Brachybacterium*, *Sphingomonas*, and *Methylobacterium* (Mariño et al., 2018). The CBB gut bacterial microbiota plays a significant role in the ability of the insect to use coffee plants as a food source by contributing to degradation of caffeine and likely to the digestion of other recalcitrant coffee seed components (Ceja-Navarro et al., 2015; Mariño et al., 2018). Knowledge about the dynamics within the gut microbial community along CBB life cycle is crucial to understand the metabolic relevance of bacteria species, the mechanisms of transmission to offspring and their potential adaptive value that allow CBB to use coffee plants as food source.

Here, we contribute to the understanding of CBB gut-associated microbiota by describing the bacterial community structure and dynamics across all insect developmental stages fed on *Coffea arabica* under field conditions in Colombian coffee crops. Gene amplicon (16S rRNA) sequencing allowed us to analyze the bacteria diversity and abundance from eggs, larvae, pupae and the adult females and males. The results presented in this study will be helpful to better understand the ecological relevance of the gut bacterial symbionts in the biology of CBB and future research for development of novel insect control strategies.

## Materials and Methods

### Insect Collection and Gut Dissection

Coffee berry borer-infested coffee berries of *Coffea arabica* var. Castillo were collected from four different sun-exposed coffee plantations at Cenicafe’s Naranjal Experimental Station (4°58′08.5″N 75°39′01.5″W) in Chinchina (Caldas, Colombia). Coffee berries were kept in the cold (∼8°C) during transportation and immediately dissected to collect life stages of CBB, including eggs; 1st instar larvae (larva-1); 2nd instar larvae (larva-2); pupae; males and females. From each coffee plantation, multiple infested berries (15–20) were dissected to collect 10 individuals for each life stage. All life stages were surface-sterilized, except for eggs, in 96% ethanol for 30 s; followed by 5.2% sodium hypochlorite for 30 s; and finally washed three times in sterile 1× PBS buffer (0.137 M NaCl; 2.7 mM KCl; 10 mM Na2HPO4; 1.8 mM KH2PO4; pH 7.4). Eggs were washed three times in sterile 1× PBS buffers. Midguts from larva-2, male and female adults were separately dissected under sterile conditions as follows: under a drop of sterile 1× PBS buffer, the anterior end (head) of larva-2 was held by a fine sterile dissecting needle and the posterior end was carefully dragged with another sterile dissecting needle until the intact midgut was exposed. Similarly, the adult pronotum was held by fine sterile forceps and the mesonotum was carefully pulled with a sterile dissecting needle until the intact midgut was exposed. Dissected guts were kept in 1× PBS at 4°C just until total DNA isolation. Surface-sterilized whole bodies of larva-1 and pupa were separately used for total DNA isolation due to limitations for gut dissection such as the small size of the larvae and the undefined internal anatomy during pupa stage.

### DNA Extraction and Sequencing

Total DNA was isolated from pools of ten midguts (from each larva-2, female and male samples) or ten whole-body (from each egg, larva-1 and pupa samples) using the DNeasy Blood & Tissue kit (*Qiagen*, Germany) and following the manufacturer protocol for gram positive bacteria. Four biological replicates per life stage, corresponding to the four coffee crop plantations above, were isolated independently. DNA integrity was checked on agarose gel and quantified using a NanoDrop 2000 (Thermo Fisher Scientific). DNA samples were vacuum dried and sent to Novogene (Sacramento, CA, United States) for PCR library amplification of the hypervariable region V3-V4 of bacterial 16S rRNA gene using primers 341F (5′-CCTAYGGGRBGCASCAG- 3′) and 806R (5′- GACTACNNGGGTATCTAAT- 3′). 16SrRNA Illumina 250PE libraries were sequenced using Novaseq platform (Illumina, San Diego, CA, EEUU).

### Processing of 16SrRNA Sequence Data

Demultiplexed raw sequences were processed using QIIME2 v. 2020.02 (Bolyen et al., 2019). Raw paired-end reads were first joined using “vsearch join-pairs” (Rognes et al., 2016). Joined reads were filtered for sequence quality using “quality-filter q-score” with default settings. Then, “deblur denoise-16S” (Amir et al., 2017) was used to remove chimeric, non-paired reads and trim sequences to 400 pb. Operational Taxonomic Units (OTUs) were *de novo* clustered at 99% of sequence identity using “vsearch cluster-features-*de-novo*” (Rognes et al., 2016). Taxonomic classification of OTUs was performed using “feature-classifier classify-sklearn” and the Greengenes database (version 13_8). OTUs with ≥ 100 reads that could not be identified to genus with Greengenes, were blasted against the NCBI 16S ribosomal RNA sequences (Bacteria and Archaea) and compared with the EzBioCloud Database (version 2020.05.13) for assignation of genera using 97% identity threshold.

### Diversity Analysis

The taxonomy and raw abundance OTU tables were exported from QIIME2 and used for taxonomic distribution and diversity analyses with MicrobiomeAnalyst^1^ (Dhariwal et al., 2017; Chong et al., 2020) through the Marker-Gene Data Profiling (MDP) module as follow: OTUs with less than four counts in at least 10% of the samples were discarded. OTU abundances were rarefied to the minimum library size and brought to the total sum scaling. Since only one replicate of pupa was available, this was excluded from the diversity analysis. Alpha-diversity was calculated with the average number of “observed OTUs” and the indices “Shannon (H’)” and “Chao1.” Statistical differences among group comparisons for each alpha-diversity index were estimated using Kruskal-Wallis test. Beta-diversity was analyzed with the Bray-Curtis distance using the rarefied OTU abundances. The permutational multivariate analysis of variance (PERMANOVA) was used to determine statistical differences in community structure as implemented in Past v.4.04 (Hammer et al., 2001). Since PERMANOVA is sensitive to the within groups dispersion (Anderson and Walsh, 2013), a test for homogeneity of multivariate dispersions (PERMDISP) was performed as implemented in MicrobiomeAnalyst. Clustering in bacterial community diversity among developmental stages were visualized with PCoA and NMDS. Taxonomic relative abundance plots were built with Past v.4.04 (Hammer et al., 2001) and heatmaps with Matrix2png^2^ (Pavlidis and Noble, 2003). Differences for bacterial taxon relative abundance among life stages were evaluated using the Kruskal-Wallis test with multiple comparison False Discovery Rate (FDR) correction using the two-stage linear step-up procedure of Benjamini, Krieger and Yekutieli (Benjamini et al., 2006), as implemented in MicrobiomeAnalyst. Shared OTUs across all developmental stages were visualized by Venn diagrams built with Jvenn^3^ (Bardou et al., 2014).

## Results

### Bacterial Diversity Across CBB Life Stages

We obtained a total of 3,543,731 raw reads of the V3-V4 region of the bacterial 16SrRNA assembled in 921,205 clean sequences (average count per sample: 43,866) from the 21 assayed samples (egg, larva, pupa, and adult) (Supplementary Figure 1). Clustering of the clean sequences at 99% identity threshold resulted in 2,723 OTUs with ≥ 2 total counts. Rarefaction curves showed a saturating number of OTUs (Supplementary Figure 2), which indicate an appropriate sequencing sampling to analyze the CBB gut bacterial diversity. Despite several attempts to obtain sequencing libraries from pupa samples, we were unable to sequence enough biological replicates for statistical analysis. Therefore, the only sample of pupa that was successfully sequenced was excluded from the diversity analysis but used for taxonomic comparisons. Thus, low-count filtering and count normalization resulted in 1,257 OTUs for taxonomic analysis and 1,761 OTUs for bacterial diversity analysis.

**FIGURE 1:**
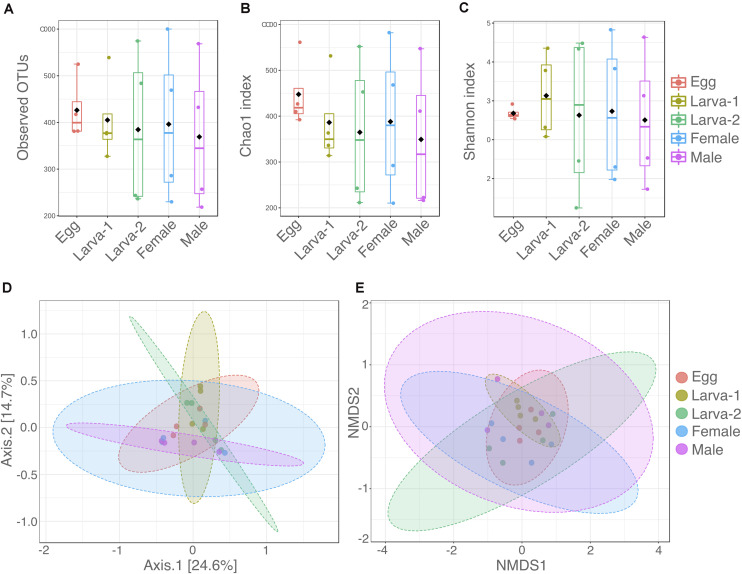
Distribution of alpha and beta diversity of gut-associated microbiota across developmental stages of *Hypothenemus hampei*. Alpha diversity was analyzed with **(A)** the number of observed OTUs; **(B)** Chao1; and **(C)** Shannon H’ indexes from four biological replicates for life stage. Beta diversity was analyzed using **(D)** principal coordinates analysis (PCoA) and **(E)** non-metric multidimensional scaling (NMDS) based on a Bray-Curtis distance matrix depicting differences in the composition of gut microbiota. Dots in panels **(D,E)** represent each sample for the life stages. Alpha and beta diversity analysis were performed using MicrobiomeAnalyst (www.microbiomeanalyst.ca/).

Diversity within each CBB life stage (Alpha-diversity) was analyzed using the number of observed OTUs, Chao1, and Shannon (H’) indexes (Supplementary Table 1 and Figures 1A–C). The average number of observed OTUs ranged from 538 to 678, however no significant differences in these numbers were found among life stages (Kruskal-Wallis test: 0.5429, *P* = 0.9692). Similarly, the average Chao1 index ranged from 598.4 to 795.3 with no significant differences among the life stages (Kruskal-Wallis test: 1.2143, *P*-value = 0.8757). The average Shannon index H’ (community diversity) ranged from 3.51 to 4.13 across CBB life stages and did not result in significant differences either (Kruskal-Wallis test: 0.1714, *P*-value = 0.9965). Differences in the microbial communities at OTU level between life stages (Beta-diversity) were analyzed by PERMANOVA and PERMDISP tests and their ordinations visualized with PCoA and NMDS (Figures 1D,E). Overall PERMANOVA (among all groups) showed differences among all the CBB life stages (*F*-value = 1.466, *P*-value = 0.0368); however, pairwise PERMANOVA (*post hoc* test, 1:1 life stage comparisons) did not allow to establish specifically which life stages were different in their bacterial communities (pairwise PERMANOVA, Bonferroni-corrected *P*-values > 0.538, uncorrected *P*-value > 0.0538 for all life stage comparisons, Supplementary Table 2). As support for the PERMANOVA analyses in this study, the PERMDISP test showed no significant differences for variation in multivariate dispersion (spread, or variability in community structure) among all life stages (PERMDISP, *F*-value = 0.43388, *P*-value = 0.78211). Additionally, distribution of bacterial communities on the PCoA and NMDS plots (Figures 1D,E) overlapped across the CBB life stages and did not showed clear separation among them.

### Overall Taxonomic Composition of the CBB Microbiota

**FIGURE 2:**
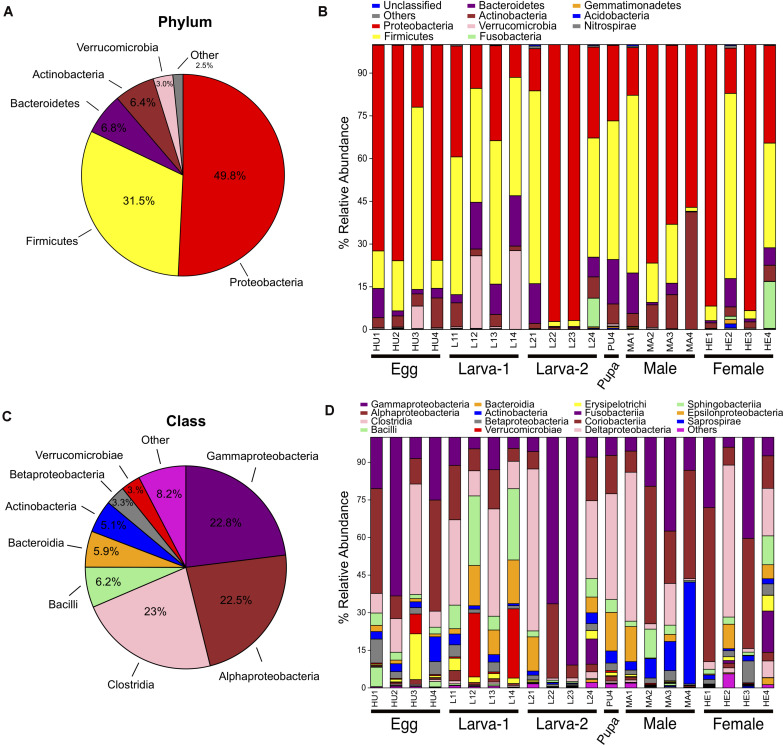
Bacterial taxonomic distribution and the Phylum and Class level within the gut-associated microbiota of *Hypothenemus hampei*. Composition at Phylum level for all samples merged **(A)** and for developmental stages **(B)**. Composition at Class level for all samples merged **(C)** and for developmental stages **(D)**.

**FIGURE 3:**
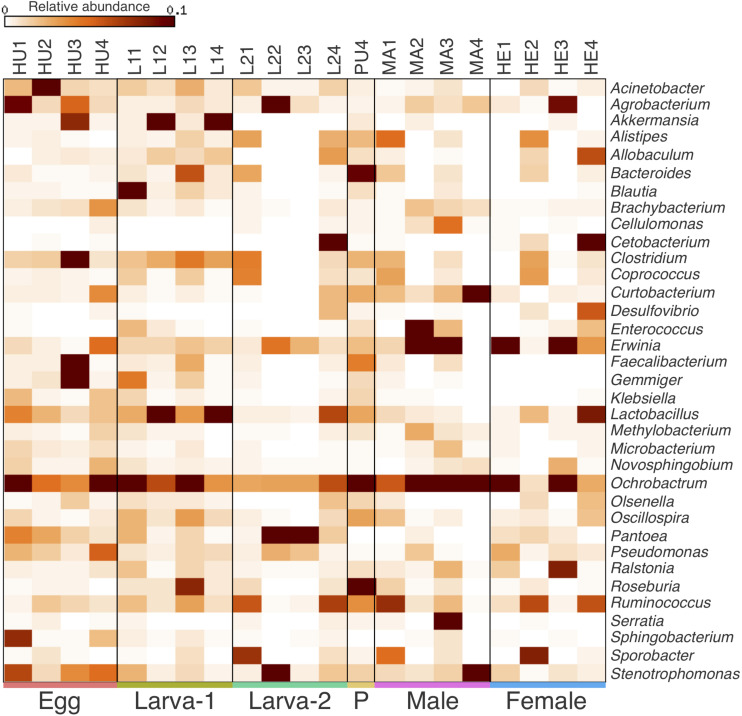
Heatmap of the relative abundance for most prevalent bacterial genera (top 35) within the gut-associated microbiota of *Hypothenemus hampei*. Columns represent the biological replicates for egg (HU1–HU4), Larva-1 (L11–L14), Larva-2 (L21–L24), pupa (PU4), male (MA1–MA4), and female (HE1–HE4). Rows represent bacteria genera.

Taxonomic assignments for OTUs were distributed across 15 bacteria phyla, 38 classes, 61 orders, 101 families, and 177 genera. Overall, the Proteobacteria (49.8%) was the most abundant Phylum, followed by Firmicutes (31.5%), Bacteroidetes (6.8%) and Actinobacteria (6.4%) (Figures 2A–C and Supplementary Figure 3). The relative abundance of each of these four top 4 bacteria phyla had no significant difference across all life stages (Kruskal-Wallis test, FDR-adjusted *P*-value >0.05, Supplementary Table 3). At the Class level, Clostridia (23%), Gammaproteobacteria (22.8%) and Alphaproteobacteria (22.5%) were collectively the most abundant groups (Figures 2B,D) and their relative abundance did not significantly change across life stages either (Kruskal-Wallis test, FDR-adjusted *P*-value >0.05, Supplementary Table 4). At the genus level, 74 genera were present at ≥0.1% relative abundance across all life stages (Figure 3 and Supplementary Table 5). From these, we considered 20 genera as the most prevalent at ≥ 1% relative abundance. In descendant order, they were: *Ochrobactrum*, *Pantoea*, *Erwinia*, *Lactobacillus*, *Acinetobacter*, *Stenotrophomonas*, *Akkermansia*, *Agrobacterium*, *Curtobacterium*, *Clostridium*, *Ruminococcus*, Bacteroides, *Roseburia*, *Gemmiger*, *Pseudomonas*, *Cetobacterium*, *Sporobacter*, *Faecalibacterium*, *Oscillospira*, and *Ralstonia*. The relative abundance of all these 20 genera did not significantly change across the CBB life cycle (Kruskal-Wallis test, FDR-adjusted *P*-value > 0.05).

### Core Microbiota of the CBB Gut

**FIGURE 4:**
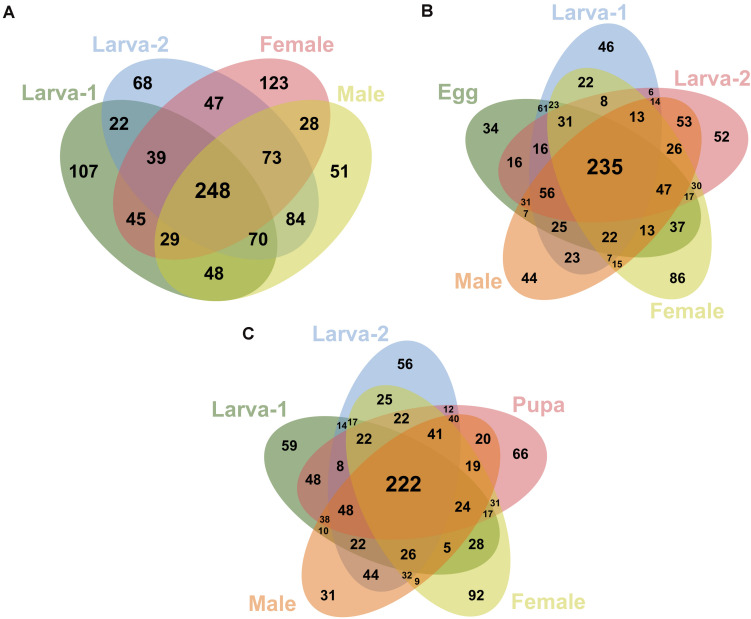
Venn diagrams showing the shared/specific bacterial OTUs (at 99% similarity) between the different developmental stages of *Hypothenemus hampei*. Comparisons are shown for the feeding life stages (larva-1, larva-2, female and male) in **(A)**; for the active-feeding life stages and egg in **(B)**; and for the active-feeding life stages and pupa in **(C)**.

We identified 248 OTUs (19.7% of all OTUs in this study) as present in ≥ 50% of the samples for each plant-feeding CBB life stage; larva-1, larva-2, male, and female (Figure 4A). From these OTUs, 235 (95%) were also detected in ≥ 50% of the egg samples (Figure 4B), whereas 222 OTUs (90%) were detected in the single pupa sample (Figure 4C). A small number of OTUs (27) were consistently detected in 100% of the life stage samples. All 20 most abundant genera listed above were shared by all life stages, including egg and pupa. Looking at the most abundant OTUs across the plant-feeding CBB life stages, we identified 20 OTUs (overall relative abundance ≥0.7%) that together account for 50% of bacterial 16SrRNA sequences found in these life stages. The DNA sequences of these abundant OTUs were compared against the bacterial 16SrRNA sequence databases in the NCBI and EzBioCloud in an attempt to assign possible bacteria species (Supplementary Table 6). Using a threshold of 98.7% sequence similarity, we identified these OTUs in decreasing order of abundance as *Ochrobactrum pseudogrignonense*; *Pantoea vagans*; *Erwinia* sp.; *Lactobacillus* sp.; *Akkermansia muciniphila*; *Curtobacterium* sp.; *Acinetobacter johnsonii*; *Agrobacterium larrymoorei*; *Stenotrophomonas* sp; *Pantoea* sp1; *Gemmiger formicilis*; *Sporobacter* sp.; *Faecalibacterium prausnitzii*; unknown *Muribaculaceae*; *Enterococcus gallinarum*; *Stenotrophomonas geniculata*; *Clostridium spiroforme*; *Serratia* sp.; *Pseudomonas* sp. and *Roseburia intestinalis*.

## Discussion

In this study, we examined the diversity and community structure of the gut-associated bacteria across the complete life cycle of CBB using a 16SrRNA gene high-throughput sequencing approach. This is the first bacteria symbiont survey that includes all CBB developmental stages (egg, 1st-instar and 2nd-instar larvae, pupae, male, and female adults) colonizing the host plant *Coffea arabica* under field conditions. In a previous study, Ceja-Navarro et al. (2015) focused on the CBB adult female and revealed a highly diverse bacteria community whose composition is significantly associated with geographic origin and host coffee species. Additionally, Mariño et al. (2018) found that CBB microbiota is also influenced by environmental conditions associated with the coffee crop system (sun-exposed vs shaded). Nonetheless, the information about the dynamic of the CBB microbiota composition along the insect life history was still missing. The identification of possible changes in the community structure of bacterial symbionts across CBB developmental stages is important for better understanding of the host-microbiota interactions. Here, we show that the general composition of the CBB gut-associated microbiota is diverse, seems to vary along the insect developmental stages and harbors a bacterial core shared with those observed in previous analysis. The existence of a bacterial core conserved across several geographic locations and within the full insect life cycle, suggest that a group of bacteria species have a symbiotic relationship with the CBB gut and potentially play important metabolic roles for food digestion and detoxification. This plausible insect-bacteria symbiotic relationship could have given CBB the adaptive capacity to use the coffee plant as a host.

We found that the overall bacteria diversity stayed unchanged across the developmental stages of CBB as revealed by the alpha diversity indexes (Observed OTUs, Chao1, and Shannon, Figures 1A–C). According to overall PERMANOVA test result, bacterial community structure at the OTU level varies in some degree across the insect life cycle. Unfortunately, pairwise PERMANOVA failed to show differences between the life stages in the 1:1 comparisons. Here, it is probable that the low number of biological replicates per life stage resulted in a low statistical power for the 1:1 comparisons. Additional observations support a degree of variability at OTU level within and across life stages, such as that less than 20% of OTUs were shared among all developmental stages and only 2% of OTUs (28 OTUs) were consistently present in 100% of the sampled replicates in the study. At higher taxonomic levels (genus level and above), overall bacteria composition seems to be more stable across the CBB life cycle; however, the apparent high variability for most taxa within each stage suggest that an analysis with larger number of samples would be necessary to generate a more precise picture of bacteria dynamics. Unlike what we observed in this study, Mariño et al. (2018) found clear significant differences between the bacterial communities from adult females and eggs in CBB. We attribute this contrasting findings likely to the fact that we sampled bacteria specifically from adult midgut tissues, whereas Mariño et al. used whole-body adults. Diverse factors can influence the composition and dynamics of bacterial community within the insect gut across life stages, including dietary shifts, changes in habitat conditions, differences in gut morphology or physicochemical properties, and gut remodeling during metamorphosis (Engel and Moran, 2013; Shukla et al., 2016; Chouaia et al., 2019; Suárez-Moo et al., 2020). In this study, we did not observe evidence for large shifts in the overall gut-associated bacteria composition across the CBB life cycle; nevertheless, we anticipate that a degree of variability appears to occur at OTU level. Based on the observations above and the fact that CBB microbiota is associated with geographic origin and host coffee species, it plausible that variation in microhabitat conditions and host-associated microbiota is shaping the CBB gut-associated bacteria (see additional discussion below).

Most attempts to screen the microbiota of the CBB pupal stage were unsuccessful in this investigation, except for a single sample that successfully amplified 16SrRNA PCR products for library preparation and sequencing. We attribute this failure to a likely low abundance of gut-associated bacteria in the CBB pupa stage. A rapid PCR screening for the 16SrRNA gene in insect DNA samples resulted in the amplification of intense DNA amplicon bands for all CBB development stages, except for pupa samples that yielded faint or none DNA amplicons (Supplementary Figure 4). Although bacteria abundance was not quantitatively tested in our analysis, we hypothesize that total abundance of gut-associated bacteria is drastically reduced during the developmental progress of the pupal stage in CBB. A similar pronounced decline of gut microbiota in the pupal stage was observed in the bark beetle *Ips pini* (Delalibera et al., 2007), the carrion beetle *Nicrophorus vespilloides* (Wang and Rozen, 2017) and in other holometabolous insects (Saraithong et al., 2017; Alfano et al., 2019). Holometabolous insects experience complete metamorphosis from distinct larva to adult. This transition involves a dramatic remodeling of external and internal anatomy during pupal development (Grimaldi et al., 2005), including a replacement of the gut, which can impact the abundance or diversity of the gut microbiota. Based on our observations, it is likely that the CBB gut bacterial symbionts undergo a similar shift of total abundance in the pupal development.

We found that Proteobacteria and Firmicutes dominate the microbiota in the CBB gut in all life stages (Figure 2A), which was similar to observations in previous studies for the CBB adult under field conditions (Mariño et al., 2018) and in other insects (Engel et al., 2012; Yun et al., 2014; Chen et al., 2018), including 13 species of the Scolytinae bark beetle *Dendroctonus* (Hernández-García et al., 2017). Among the most prevalent bacteria genera in our study, there are members of the most common taxa found in the microbiomes of arthropods, such as *Ochrobactrum*, *Pantoea*, *Erwinia*, *Acinetobacter*, *Stenotrophomonas*, *Agrobacterium*, and *Pseudomonas* (Degli Esposti and Martinez Romero, 2017). From genera at ≥ 0.1% relative abundance, *Ochrobactrum*, *Pantoea*, *Erwinia*, *Acinetobacter*, *Agrobacterium*, *Pseudomonas*, *Brachybacterium*, *Methylobacterium*, and *Sphingomonas* have been consistently found in adult female CBBs from other coffee-growing regions from Africa, America and Asia (Ceja-Navarro et al., 2015; Mariño et al., 2018), therefore we can consider these as the core gut microbiota of *H. hampei*, as suggested by previous research (Mariño et al., 2018). At the genus level, the bacterial community we observed in the CBB adults in Colombia does not differ drastically from the community found in Puerto Rico (Mariño et al., 2018) in terms of the presence of most prevalent taxa. A similar observation results when comparing the egg microbiota from these two locations. However, few bacteria genera showed differences at the relative abundances in the adult, as observed for *Ochrobactrum* and *Erwinia* which dominated the bacterial community in CBB adults in Colombia; while *Pantoea* and *Pseudomonas* dominated the community in Puerto Rico. These differences in abundance of prevalent bacteria; along with other differences for presence/absence for low-abundance genera, indicate geographical variations for the CBB-associated bacterial community, likely influenced by differences in environmental conditions and/or host-plant associated microbiota.

Several CBB gut bacteria strains of *Pantoea*, *Pseudomonas*, *Ochrobactrum*, *Stenotrophomonas*, *Enterobacter*, *Microbacterium*, *Novosphingobium*, and *Brachybacterium* were previously isolated as capable of subsisting on caffeine as a sole carbon and nitrogen source (Ceja-Navarro et al., 2015). In the same study, it was demonstrated *in vivo* that *Pseudomonas fulva* degrades caffeine within the insect gut. Recently, it was shown that CBB-associated *P. fulva* and other four *Pseudomona* species contains a full gene complement for caffeine metabolism, while other additional sixteen bacteria species contain partial gene complements for the same process (Vega et al., 2021). Caffeine degradation capability has been shown for other strains of *Stenotrophomonas*, *Serratia*, *Acinetobacter*, *Klebsiella*, *Rhodococcus*, and *Methylobacterium* as well (Madyastha and Sridhar, 1998; Yamaoka and Mazzafera, 1998; Iswanto et al., 2019). Bacteria in the genus *Pseudomonas* were shown to be highly abundant (21–25%) in CBB females from coffee plots in Puerto Rico (Mariño et al., 2018) but not in our study (1.1% in the CBB female sample). Similarly, Ceja-Navarro et al. (2015) observed high variation for *Pseudomonas* relative abundance among CBB microbiotas from several coffee-producing locations; ranging from extreme low abundance in Indonesia to almost 20% in India. This observation and the possibility that some of the bacterial genera within the CBB gut may possess caffeine-degrading capabilities, suggest that not only *Pseudomonas* but a consortium of several gut-associated bacteria species with metabolic redundancy could have a role in caffeine detoxification and offer an ecological advantage to this coffee pest.

Bacteria in the genera *Pantoea*, *Erwinia*, *Serratia*, and *Klebsiella* have been isolated from several phytophagous insects as gut symbionts with capacities for plant material digestion (Bashir et al., 2013; Shil et al., 2014; Dantur et al., 2015; Bozorov et al., 2019). Other bacteria in the genera *Pseudomonas* and *Stenotrophomonas*, along with *Serratia* and *Methylobacterium*, were also isolated from the bark beetles *Dendroctonus rhizophagus* and *D. armandi* showing cellulolytic activity (Morales-Jiménez et al., 2012; Hu et al., 2014; Briones-Roblero et al., 2017). Within the CBB gut, we found seven abundant OTUs associated with *Pantoea*, *Erwinia*, *Stenotrophomonas* and *Serratia* consistently across larvae and adults (Supplementary Table 6). The same genera were also abundant in the CBB adult gut microbiota from other geographic locations (Ceja-Navarro et al., 2015; Mariño et al., 2018). It is likely that these bacteria play similar physiological roles in the CBB gut as those found for gut-associated symbionts in other phytophagous insects. Future research involving bacterial metagenomic and metatranscriptomic analyses will be necessary in order to obtain a better picture of the physiological roles of the gut microbiota in the biology of the CBB and its contribution in the digestion of plant cell structural components.

Our results indicate that the CBB egg harbors a microbiota as complex as the larvae or the adults, having probably similar diversity and structure (Figures 1A–E). Most bacteria OTUs found in the egg (∼70%) were also observed in the 1st-instar larva. This observation may indicate that most CBB gut bacteria could be transmitted vertically to newborns via egg. Potential indications of transovarial transmission of two gut-associated *Pseudomonas* species from the CBB mother to its offspring was recently presented (Vega et al., 2021); however, further research is necessary to test whether other bacteria species could be transmitted via egg by internal inoculation and/or external shell inoculation. From the total bacterial OTUs detected in the egg, 70% to 60% were commonly found in larvae and adults of CBB, including OTUs for all members of the bacterial core genera proposed above. A similar result was obtained previously (Mariño et al., 2018), where eggs and adults of CBB shared 60% of the bacteria genera, including also members of the core microbiota. Despite these observations, it cannot be ruled out the possibility that some bacteria may be acquired from the host plant throughout feeding of the developing CBB larvae and adults. This idea is based on the fact that several of the bacterial genera observed in the CBB gut have been detected as endophytic microbes in the coffee berry tissues, including *Pantoea*, *Pseudomonas*, *Stenotrophomonas*, *Bacillus*, and *Serratia* among the most abundant (Vega et al., 2005; Nunes and de Melo, 2006; Vaughan et al., 2015). Since we did not screen the endophytic microbiota in the coffee plant host at the same time as insect-associated bacteria was analyzed in this study, it is still necessary to investigate whether there is a strong influence of the plant host’s microbial environment on the microbiota of the CBB digestive tract. Future research will need to elucidate the metabolic contribution and the mechanisms that maintain an insect gut bacterial core throughout the life cycle of the insect and through generations. It is possible that members of the CBB gut core bacteria were acquired originally during the adaptation process of CBB to subsist on coffee plants and later established as gut-associated symbionts.

## Conclusion

The findings presented here improve the knowledge concerning the dynamics of the gut microbial community associated with the CBB gut during the insect life history. Our results indicate that the overall bacterial community composition is highly diverse, variable within each life stage and appears to vary at some degree across the developmental stages of CBB. The persistent detection of genera *Pantoea*, *Erwinia*, *Acinetobacter*, *Ochrobactrum*, *Agrobacterium*, *Pseudomonas*, *Brachybacterium*, *Methylobacterium*, and *Sphingomonas* as members of the microbial core suggest that these bacteria must play significant roles in the ecology of CBB and its interactions with the host coffee plant. The CBB gut-associated core bacteria can serve as targets for future functional analyses in order to establish the physiological contributions of the insect microbiome and to develop novel pest control strategies.

## Data Availability Statement

The raw Illumina sequences generated for this study can be found in the NCBI (https://www.ncbi.nlm.nih.gov/) under BioProject number PRJNA682196.

## Author Contributions

LN-E designed the project. FM-A and LN-E conducted the research. FM-A, LN-E, and TG-H analyzed the data. LN-E and FM-A wrote the manuscript. TG-H, CG, and PB made conceptual contributions and contributed to writing and editing the manuscript. All authors contributed to the article and approved the submitted version.

## Conflict of Interest

The authors declare that the research was conducted in the absence of any commercial or financial relationships that could be construed as a potential conflict of interest.

## Footnotes

1 https://www.microbiomeanalyst.ca/

2 https://matrix2png.msl.ubc.ca/

3 http://jvenn.toulouse.inra.fr/
