# Supplementary material for: Structure and Dynamics of the Gut Bacterial Community Across the Developmental Stages of the Coffee Berry Borer, Hypothenemus hampei
Source: Front Microbiol. 2021 Jul 1;12:639868. doi: 10.3389/fmicb.2021.639868 (PMC8323054; doi:10.3389/fmicb.2021.639868)
Supplement: Supplementary file 1 [file Data_Sheet_1.docx]

**Supplementary Data**

**Supplementary Tables:**

**Supplementary Table S1**. Alpha diversity indices of bacterial community at different life stages of coffee berry borer.

| **Life stage** | **Sequences** | **Observed OTUs** | **Chao1** | **Shannon (H’)** |
| --- | --- | --- | --- | --- |
| Egg | 46,821 ± 9,560 | 652.5  ± 136 | 795.3 ± 154.6 | 3.68 ± 0.17 |
| 1st-instar larva | 29,501 ± 10,692 | 610.3 ± 184.5 | 672.4 ± 198 | 4.13 ± 1.11 |
| 2nd-instar larva | 45,640 ± 18,198 | 569.3 ± 324.1 | 629.7 ± 329.1 | 3.63 ± 2.12 |
| Pupa^*^ | 27,270 | 678 | - | - |
| Adult male | 45,612 ± 21,458 | 538.3 ± 324.6 | 598.4 ± 319.9 | 3.51 ± 1.74 |
| Adult female | 54,646 ± 18,764 | 592.5  ± 339.8 | 676.2 ± 336.1 | 3.73 ± 1.89 |

*One sample

**Supplementary Table S2.** Results of pairwise PERMANOVA testing for differences in beta diversity between CBB developmental stages based on the Bray-Curtis distance. Bonferroni-corrected and uncorrected P-values are presented in the upper right and lower left portions of the table, respectively.

|  | **Egg** | **Larva-1** | **Larva-2** | **Female** | **Male** |
| --- | --- | --- | --- | --- | --- |
| **Egg** |  | 1 | 0.843 | 1 | 1 |
| **Larva-1** | 0.1717 |  | 0.538 | 0.841 | 0.568 |
| **Larva-2** | 0.091 | 0.0599 |  | 1 | 1 |
| **Female** | 0.2862 | 0.0817 | 0.4272 |  | 1 |
| **Male** | 0.1729 | 0.0582 | 0.1976 | 0.6863 |  |

**Supplementary Table S3**. Kruskal-Wallis test results for differences at the Phylum level among CBB developmental stages.

| **Phylum** | **P-values** | **FDR*** | **Statistics** |
| --- | --- | --- | --- |
| Verrucomicrobia | 0.012891 | 0.25781 | 12.691 |
| Fusobacteria | 0.062323 | 0.62323 | 8.9515 |
| Actinobacteria | 0.10379 | 0.69196 | 7.6857 |
| TM7 | 0.32838 | 0.99544 | 4.6215 |
| Bacteroidetes | 0.34938 | 0.99544 | 4.4429 |
| Chlorobi | 0.38814 | 0.99544 | 4.1343 |
| Proteobacteria | 0.39076 | 0.99544 | 4.1143 |
| TM6 | 0.42319 | 0.99544 | 3.875 |
| Spirochaetes | 0.53033 | 0.99544 | 3.1667 |
| Cyanobacteria | 0.689 | 0.99544 | 2.2549 |
| Tenericutes | 0.73107 | 0.99544 | 2.0255 |
| WS3 | 0.73116 | 0.99544 | 2.025 |
| Firmicutes | 0.75414 | 0.99544 | 1.9 |
| Chloroflexi | 0.8803 | 0.99544 | 1.1866 |
| AD3 | 0.88714 | 0.99544 | 1.1445 |
| Acidobacteria | 0.91728 | 0.99544 | 0.95 |
| Gemmatimonadetes | 0.97054 | 0.99544 | 0.52977 |
| Deferribacteres | 0.97691 | 0.99544 | 0.46404 |
| Nitrospirae | 0.99544 | 0.99544 | 0.19732 |

*False Discovery rate-adjusted P-value

**Supplementary Table S4**. Kruskal-Wallis test results for differences at the Class level among CBB developmental stages.

| **Class** | **P-values** | **FDR*** | **Statistics** |
| --- | --- | --- | --- |
| Verrucomicrobiae | 0.012891 | 0.6961 | 12.691 |
| Actinobacteria | 0.034823 | 0.79012 | 10.357 |
| Fusobacteriia | 0.062323 | 0.79012 | 8.9515 |
| Sphingobacteriia | 0.082039 | 0.79012 | 8.2741 |
| Flavobacteriia | 0.11793 | 0.79012 | 7.3625 |
| Gitt_GS_136 | 0.12202 | 0.79012 | 7.2757 |
| Betaproteobacteria | 0.12639 | 0.79012 | 7.1857 |
| Bacilli | 0.13439 | 0.79012 | 7.0286 |
| Erysipelotrichi | 0.14921 | 0.79012 | 6.7587 |
| Sva0725 | 0.19034 | 0.79012 | 6.1204 |
| Cytophagia | 0.23413 | 0.79012 | 5.5644 |
| iii1_8 | 0.27348 | 0.79012 | 5.1375 |
| Bacteroidia | 0.30075 | 0.79012 | 4.8714 |
| Coriobacteriia | 0.30188 | 0.79012 | 4.8608 |
| Epsilonproteobacteria | 0.32069 | 0.79012 | 4.6892 |
| TM7_3 | 0.32838 | 0.79012 | 4.6215 |
| SJA_28 | 0.38814 | 0.79012 | 4.1343 |
| Alphaproteobacteria | 0.40989 | 0.79012 | 3.9714 |
| SJA_4 | 0.42319 | 0.79012 | 3.875 |
| Anaerolineae | 0.44622 | 0.79012 | 3.7131 |
| Deltaproteobacteria | 0.44813 | 0.79012 | 3.7 |
| Saprospirae | 0.45272 | 0.79012 | 3.6685 |
| S085 | 0.45325 | 0.79012 | 3.6649 |
| Gammaproteobacteria | 0.46071 | 0.79012 | 3.6143 |
| JG37_AG_4 | 0.46161 | 0.79012 | 3.6082 |
| Chloracidobacteria | 0.50035 | 0.79012 | 3.3545 |
| RF3 | 0.51153 | 0.79012 | 3.2836 |
| Ellin6529 | 0.52048 | 0.79012 | 3.2276 |
| Acidobacteria_6 | 0.52962 | 0.79012 | 3.1711 |
| BPC102 | 0.52962 | 0.79012 | 3.1711 |
| C0119 | 0.52962 | 0.79012 | 3.1711 |
| Chloroflexi | 0.52962 | 0.79012 | 3.1711 |
| Thermomicrobia | 0.52962 | 0.79012 | 3.1711 |
| Brevinematae | 0.53033 | 0.79012 | 3.1667 |
| EC1113 | 0.53033 | 0.79012 | 3.1667 |
| PAUC37f | 0.53033 | 0.79012 | 3.1667 |
| DA052 | 0.54138 | 0.79012 | 3.0991 |
| 4C0d_2 | 0.689 | 0.93015 | 2.2549 |
| TK10 | 0.689 | 0.93015 | 2.2549 |
| PRR_12 | 0.73116 | 0.963 | 2.025 |
| RB25 | 0.77894 | 0.99544 | 1.7647 |
| Mollicutes | 0.79541 | 0.99544 | 1.6742 |
| Thermoleophilia | 0.88686 | 0.99544 | 1.1463 |
| ABS_6 | 0.90988 | 0.99544 | 0.99943 |
| Gemmatimonadetes | 0.93384 | 0.99544 | 0.83391 |
| Solibacteres | 0.93567 | 0.99544 | 0.8206 |
| MB_A2_108 | 0.94241 | 0.99544 | 0.77007 |
| Acidobacteriia | 0.95299 | 0.99544 | 0.68644 |
| Clostridia | 0.96619 | 0.99544 | 0.57143 |
| Acidimicrobiia | 0.97155 | 0.99544 | 0.5197 |
| Deferribacteres | 0.97691 | 0.99544 | 0.46404 |
| Gemm_1 | 0.98349 | 0.99544 | 0.38746 |
| Nitrospira | 0.99544 | 0.99544 | 0.19732 |

*False Discovery rate-adjusted P-value

**Supplementary Table S5**. Overall relative abundance for most prevalent genera (top 50) across CBB developmental stages. Overall relative abundance corresponds to the combination of all replicates per life stage.

| **Genus** | **Egg** | **Female** | **Larva-1** | **Larva-2** | **Male** | **Pupa** |
| --- | --- | --- | --- | --- | --- | --- |
| *Ochrobactrum* | 0.1734 | 0.2227 | 0.0768 | 0.0382 | 0.2576 | 0.1034 |
| *Pantoea* | 0.0234 | 0.0073 | 0.0100 | 0.3021 | 0.0019 | 0.0003 |
| *Erwinia* | 0.0159 | 0.1552 | 0.0146 | 0.0214 | 0.0895 | 0.0198 |
| *Lactobacillus* | 0.0216 | 0.0246 | 0.1490 | 0.0194 | 0.0063 | 0.0283 |
| *Acinetobacter* | 0.1468 | 0.0048 | 0.0138 | 0.0098 | 0.0037 | 0.0060 |
| *Stenotrophomonas* | 0.0385 | 0.0077 | 0.0105 | 0.0615 | 0.0396 | 0.0140 |
| *Akkermansia* | 0.0218 | 0.0010 | 0.1320 | 0.0006 | 0.0012 | 0.0085 |
| *Curtobacterium* | 0.0124 | 0.0042 | 0.0037 | 0.0060 | 0.1091 | 0.0254 |
| *Ruminococcus* | 0.0129 | 0.0325 | 0.0192 | 0.0324 | 0.0262 | 0.0368 |
| *Clostridium* | 0.0725 | 0.0109 | 0.0285 | 0.0138 | 0.0088 | 0.0226 |
| *Agrobacterium* | 0.0426 | 0.0234 | 0.0077 | 0.0664 | 0.0115 | 0.0032 |
| *Roseburia* | 0.0040 | 0.0030 | 0.0260 | 0.0043 | 0.0060 | 0.1105 |
| *Bacteroides* | 0.0048 | 0.0117 | 0.0196 | 0.0097 | 0.0069 | 0.0870 |
| *Faecalibacterium* | 0.0279 | 0.0008 | 0.0108 | 0.0027 | 0.0046 | 0.0467 |
| *Oscillospira* | 0.0069 | 0.0088 | 0.0191 | 0.0066 | 0.0085 | 0.0276 |
| *Gemmiger* | 0.0449 | 0.0004 | 0.0155 | 0.0015 | 0.0012 | 0.0110 |
| *Pseudomonas* | 0.0242 | 0.0105 | 0.0107 | 0.0132 | 0.0063 | 0.0025 |
| *Alistipes* | 0.0025 | 0.0086 | 0.0081 | 0.0138 | 0.0133 | 0.0203 |
| *Cetobacterium* | 0.0009 | 0.0392 | 0.0002 | 0.0227 | 0.0013 | 0.0019 |
| *Sporobacter* | 0.0035 | 0.0188 | 0.0035 | 0.0194 | 0.0136 | 0.0041 |
| *Ralstonia* | 0.0057 | 0.0247 | 0.0107 | 0.0034 | 0.0088 | 0.0088 |
| *Enterococcus* | 0.0013 | 0.0077 | 0.0084 | 0.0020 | 0.0302 | 0.0101 |
| *Allobaculum* | 0.0058 | 0.0223 | 0.0123 | 0.0075 | 0.0020 | 0.0087 |
| *Coprococcus* | 0.0043 | 0.0100 | 0.0103 | 0.0119 | 0.0092 | 0.0111 |
| *Blautia* | 0.0029 | 0.0010 | 0.0318 | 0.0015 | 0.0019 | 0.0113 |
| *Serratia* | 0.0022 | 0.0008 | 0.0014 | 0.0024 | 0.0385 | 0.0036 |
| *Brachybacterium* | 0.0162 | 0.0033 | 0.0050 | 0.0026 | 0.0109 | 0.0031 |
| *Olsenella* | 0.0065 | 0.0074 | 0.0066 | 0.0042 | 0.0023 | 0.0117 |
| *Methylobacterium* | 0.0063 | 0.0030 | 0.0044 | 0.0025 | 0.0101 | 0.0100 |
| *Novosphingobium* | 0.0126 | 0.0071 | 0.0049 | 0.0029 | 0.0059 | 0.0025 |
| *Sphingobacterium* | 0.0254 | 0.0012 | 0.0041 | 0.0018 | 0.0026 | 0.0006 |
| *Klebsiella* | 0.0110 | 0.0007 | 0.0058 | 0.0013 | 0.0008 | 0.0147 |
| *Kineothrix* | 0.0020 | 0.0032 | 0.0052 | 0.0028 | 0.0024 | 0.0148 |
| *Desulfovibrio* | 0.0022 | 0.0144 | 0.0009 | 0.0056 | 0.0010 | 0.0038 |
| *Bacillus* | 0.0033 | 0.0009 | 0.0107 | 0.0030 | 0.0020 | 0.0061 |
| *Microbacterium* | 0.0083 | 0.0023 | 0.0032 | 0.0017 | 0.0071 | 0.0015 |
| *Cellulomonas* | 0.0015 | 0.0011 | 0.0007 | 0.0018 | 0.0145 | 0.0042 |
| *Xenophilus* | 0.0142 | 0.0003 | 0.0034 | 0.0015 | 0.0010 | 0.0000 |
| *Butyrivibrio* | 0.0001 | 0.0000 | 0.0009 | 0.0002 | 0.0006 | 0.0174 |
| *Romboutsia* | 0.0022 | 0.0034 | 0.0100 | 0.0012 | 0.0011 | 0.0014 |
| *Vibrio* | 0.0003 | 0.0018 | 0.0053 | 0.0053 | 0.0049 | 0.0000 |
| *Brevundimonas* | 0.0035 | 0.0012 | 0.0018 | 0.0015 | 0.0025 | 0.0069 |
| *Escherichia_Shigella* | 0.0030 | 0.0025 | 0.0044 | 0.0009 | 0.0012 | 0.0053 |
| *Adlercreutzia* | 0.0022 | 0.0046 | 0.0048 | 0.0031 | 0.0008 | 0.0016 |
| *Sphingobium* | 0.0052 | 0.0008 | 0.0059 | 0.0006 | 0.0022 | 0.0006 |
| *Enhydrobacter* | 0.0098 | 0.0008 | 0.0025 | 0.0006 | 0.0008 | 0.0000 |
| *Sutterella* | 0.0014 | 0.0003 | 0.0020 | 0.0013 | 0.0011 | 0.0080 |
| *Helicobacter* | 0.0010 | 0.0060 | 0.0004 | 0.0029 | 0.0007 | 0.0017 |
| *Delftia* | 0.0065 | 0.0013 | 0.0019 | 0.0009 | 0.0007 | 0.0008 |
| *Fusicatenibacter* | 0.0009 | 0.0010 | 0.0013 | 0.0029 | 0.0024 | 0.0036 |
| Other | 0.1311 | 0.2686 | 0.2499 | 0.2527 | 0.2132 | 0.2459 |

**Supplementary Table S6**. Overall most prevalent OTUs (top 20) within the CBB gut microbiota in larva and adult stages.

| **Rank** | **Relative abundance (%)** | **Taxonomic annotation** | | |
| --- | --- | --- | --- | --- |
|  |  | **Greengenes** | **Top-hit BLASTn**  **(sequence similarity)** | **Top-hit EzBioCloud^#^**  **(sequence similarity)** |
| 1 | 15.1 | *Ochrobactrum* | *Ochrobactrum pseudogrignonense* (100%) | *Ochrobactrum pseudogrignonense* (100%) |
| 2 | 5.1 | *Pantoea* | *Pantoea vagans* (100%) | *Pantoea vagans* (100%) |
| 3 | 4.3 | *Erwinia* | *Erwinia uzenensis* (98%) | *Erwinia uzenensis* (98.25%) |
| 4 | 3.3 | *Lactobacillus* | *Lactobacillus animalis* (100%), *L. murinus* (100%), *L. apodemi* (100%) | *Ligilactobacillus* *animalis* (100%) |
| 5 | 3.0 | *Akkermansia muciniphila* | *Akkermansia muciniphila* (100%) | *Akkermansia muciniphila* (100%) |
| 6 | 2.7 | *Curtobacterium* | *Curtobacterium oceanosedimentum* (99%) | *Curtobacterium flaccumfaciens* (100%) |
| 7 | 2.5 | *Acinetobacter johnsonii* | *Acinetobacter johnsonii* (99.5%) | *Acinetobacter* DQ532297_s (99.25%) |
| 8 | 2.2 | *Agrobacterium* | *Agrobacterium larrymoorei* (100%) | *Agrobacterium larrymoorei* (100%) |
| 9 | 2.1 | *Stenotrophomonas* | *Stenotrophomonas* *pavanii* (98.5%), *S. maltophilia* (98.5%) | *Stenotrophomonas* CP026001_s (100%) |
| 10 | 1.5 | *Enterobacteriaceae* | *Erwinia persicina* (99%) | *Pantoea* JTJJ_s (99.75%) |
| 11 | 1.2 | *Gemmiger formicilis* | *Gemmiger formicilis* (100%) | *Gemmiger formicilis* (100%) |
| 12 | 1.1 | *Ruminococcaceae* | *Intestinimonas butyriciproducens* (92.1%) | *Sporobacter* EF445147_s (100%) |
| 13 | 1.1 | *Faecalibacterium prausnitzii* | *Faecalibacterium prausnitzii* (99%) | *Faecalibacterium prausnitzii* (99.75) |
| 14 | 1.1 | *Bacteroidales* | *Muribaculum intestinale* (86.4%) | Unknown *Muribaculaceae* (99.25%) |
| 15 | 0.99 | *Enterococcus* | *Enterococcus gallinarum* (100%) | *Enterococcus gallinarum* (100%) |
| 16 | 0.95 | *Stenotrophomonas geniculata* | [*Pseudomonas*] *geniculata* (100%) | *Stenotrophomonas* *geniculata* (100%) |
| 17 | 0.90 | *Clostridium spiroforme* | *[Clostridium] spiroforme* (100%) | *Clostridium spiroforme* (100%) |
| 18 | 0.88 | *Serratia marcescens* | *Serratia* *nematodiphila* (100%) | *Serratia marcescens* (99.75%) |
| 19 | 0.76 | *Pseudomonas* | *Pseudomonas fulva* (99.25%) | *Pseudomonas kribbensis* (99.75%) |
| 20 | 0.74 | *Roseburia* | *Roseburia intestinalis* (100%) | *Roseburia intestinalis* (100%) |

^#^ EzBioCloud database: [http://ezbiocloud.net](http://ezbiocloud.net/). Threshold for species identification: >98.7%

**Supplementary Figures:**


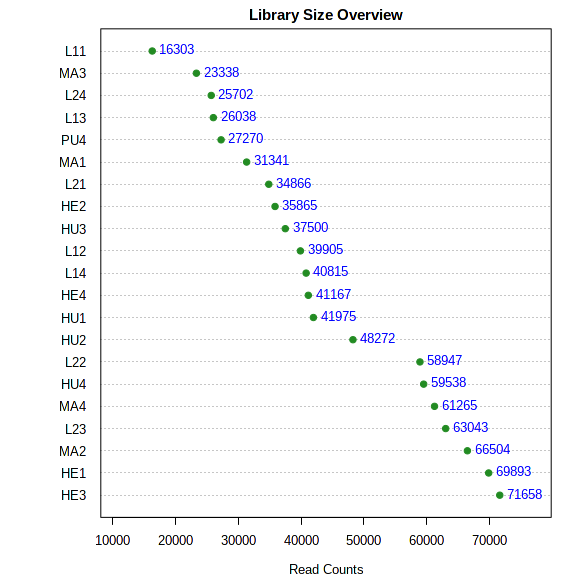


**Supplementary Figure S1**. Distribution of the total number of sequences (read counts) for all samples analyzed in this study.


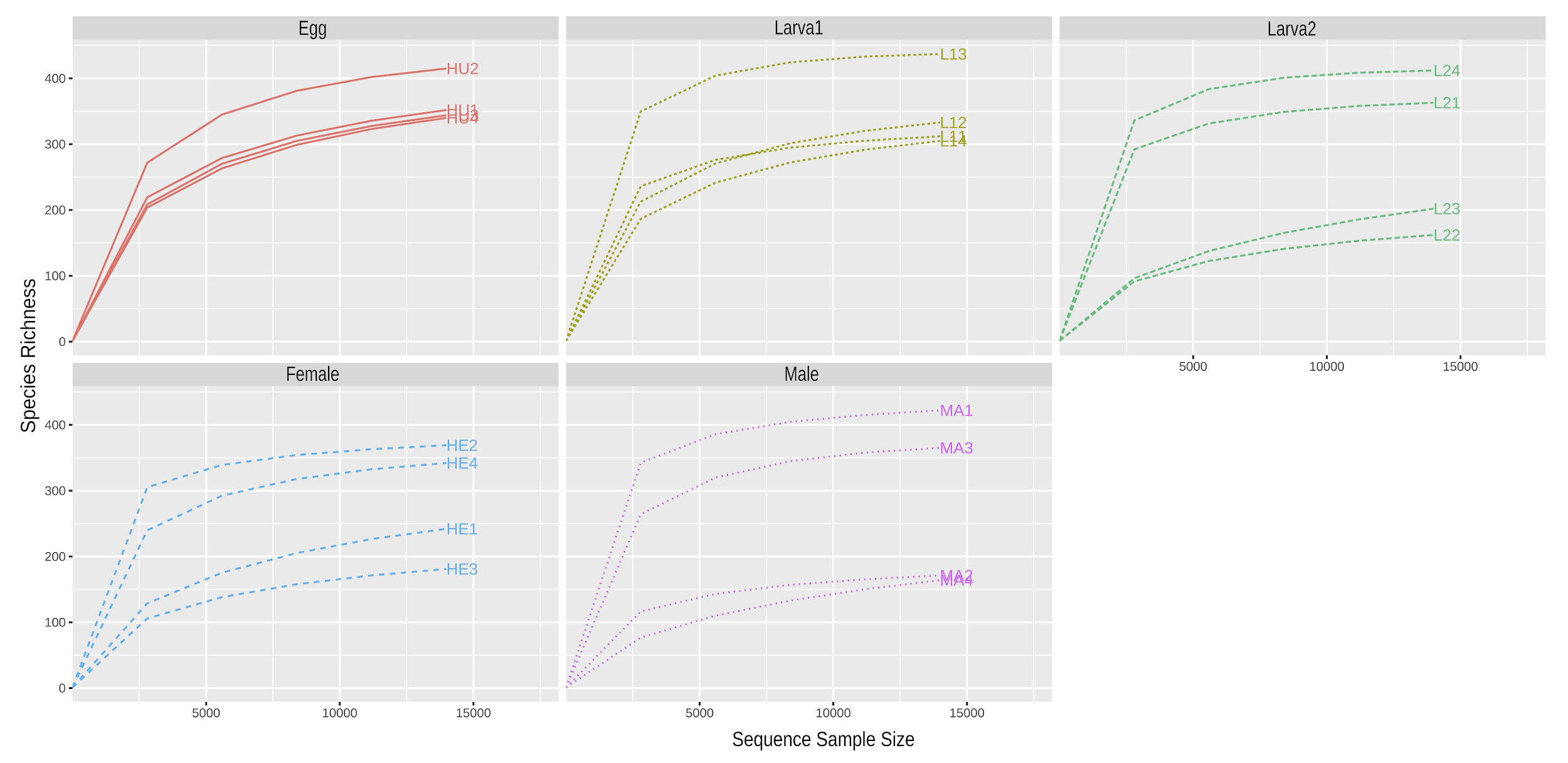


**Supplementary Figure S2**. Rarefaction curves for samples of CBB developmental stages based on the number of OTUs. Analyses and plots were produced by MicrobiomeAnalyst (<https://www.microbiomeanalyst.ca/>).


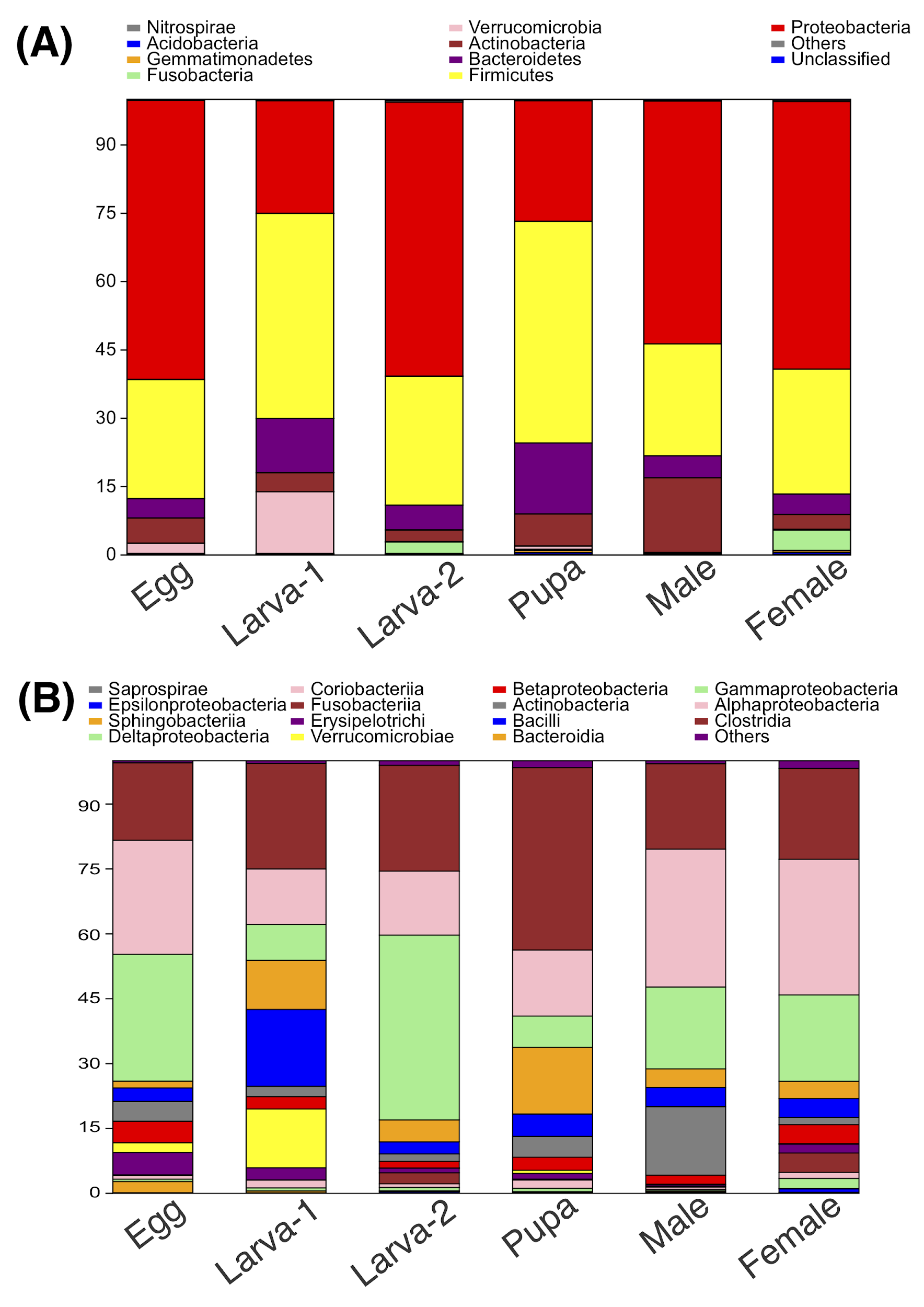


**Supplementary Figure S3**. Bacterial taxonomic distribution and the Phylum and Class level within the gut-associated microbiota of *Hypothenemus hampei*. Bacterial composition across all insect developmental stages at Phylum and Class levels are shown in (A) and (B), respectively.


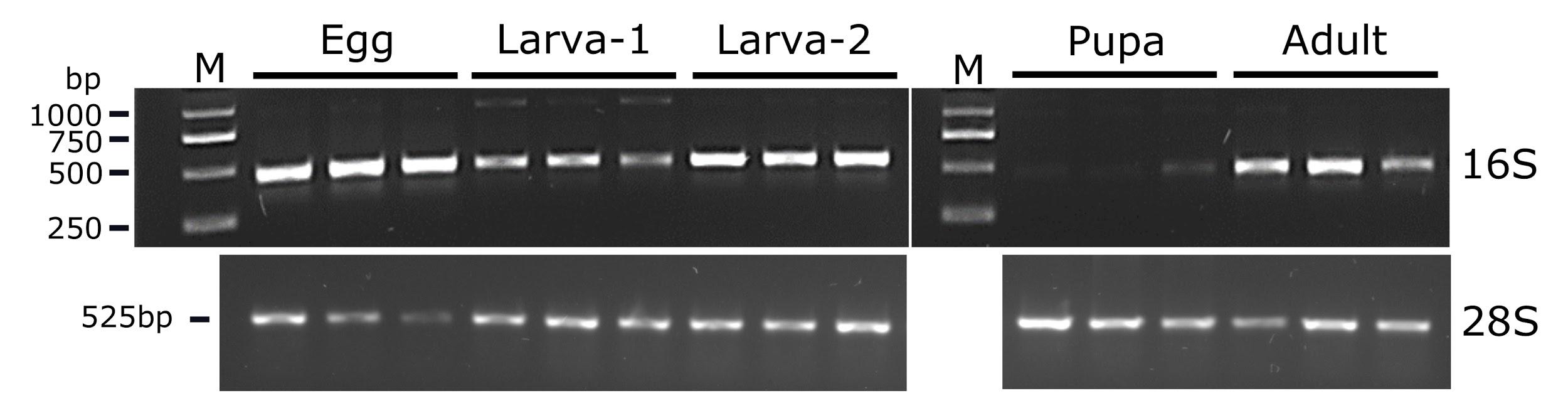


**Supplementary Figure S4**. Molecular screening for detection of bacterial 16S rRNA sequences across CBB life stages. Conventional PCR reactions were performed using total DNA from CBB life stages as template (100ng/20uL reaction) and the primers 341F (5’-CCTAYGGGRBGCASCAG-3’) and 806R (5’-GGACTACNNGGGTATCTAAT-3’) for 16S rRNA genes. As PCR control, 20ng/20uL reaction of total DNA from each life stage was used as template to amplify a 525bp fragment of the 28S rRNA gene using primers 28sF3633 (5’-TACCGTGAGGGAAAGTTGAAA-3’) and 28sR4076: (5’-AGACTCCTTGGTCCGTGTTT-3’). The cycling parameters were: initial denaturation at 94°C for 2 min, followed by 35 cycles of denaturation at 94 °C for 30 sec, annealing at 55 °C for 30 sec and extension at 72 °C for 40 sec, and a final extension at 72 °C for 2 min.
